# Phosphatidylinositol 3‐kinase‐δ controls endoplasmic reticulum membrane fluidity and permeability in fungus‐induced allergic inflammation in mice

**DOI:** 10.1111/bph.14917

**Published:** 2020-01-27

**Authors:** Hwa‐Young Lee, Geum‐Hwa Lee, Hyung‐Ryong Kim, Yong‐Chul Lee, Han‐Jung Chae

**Affiliations:** ^1^ Department of Pharmacology and Institute of New Drug Development Chonbuk National University Medical School Jeonju Republic of Korea; ^2^ Non‐Clinical Evaluation Center, Biomedical Research Institute Chonbuk National University Hospital Chonbuk Republic of Korea; ^3^ College of Dentistry, Institute of Tissue Regeneration Engineering (ITREN) Dankook University Cheonan Republic of Korea; ^4^ Department of Internal Medicine Chonbuk National University Medical School Chonbuk Republic of Korea

## Abstract

**Background and Purpose:**

Phosphatidylinositol 3‐kinase (PI3K), especially PI3K‐δ, and endoplasmic reticulum (ER) stress play important roles in refractory asthma induced by the fungus *Aspergillus fumigatus* through mechanisms that are not well understood. Here we have investigated these mechanisms, using BEAS‐2B human bronchial epithelial cells and a mouse model of *A. fumigatus*‐induced allergic lung inflammation.

**Experimental Approach:**

A selective PI3K‐δ inhibitor, IC87114, and an ER folding chaperone, 4‐phenylbutyric acid (4‐PBA), were applied to a model of *A. fumigatus*‐induced asthma in female C57BL/6 mice. The therapeutic potential of IC87114 and 4‐PBA was assessed in relevant primary cell, tissue, and disease models, using immunohistochemistry, western blotting and assessment of ER redox state and membrane fluidity.

**Key Results:**

Treatment with IC87114 or 4‐PBA alleviated pulmonary inflammation and airway remodelling and reduced ER stress and inflammation‐associated intra‐ER hyperoxidation, disrupting protein disulfide isomerase (PDI) chaperone activity. IC87114 and 4‐PBA also reversed changes in ER membrane fluidity and permeability and the resultant mitochondrial hyperactivation (i.e., Ca^2+^ accumulation) under hyperoxidation, thereby restoring the physiological state of the ER and mitochondria. These compounds also abolished mitochondria‐associated ER membrane (MAM) formation caused by the physical contact between these subcellular organelles.

**Conclusion and Implications:**

PI3K‐δ and ER stress mediate *A. fumigatus*‐induced allergic lung inflammation by altering the ER redox state, PDI chaperone function, and ER membrane fluidity and permeability and by amplifying ER signalling to mitochondria through MAM formation. Thus, therapeutic strategies that target the PI3K‐δ–ER stress axis could be an effective treatment for allergic asthma caused by fungi.

## Graphical abstract

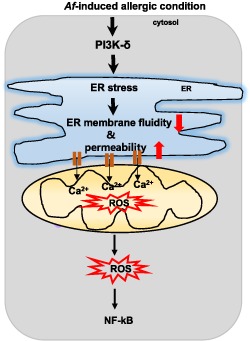

**Abbreviations**
4‐HNE: 4‐hydroxynonenal
4‐PBA: 4‐phenylbutyric acid
AHR: airway hyper‐responsiveness
BALF: bronchoalveolar lavage fluid
CHOP: CCAAT‐enhancer‐binding protein homologous protein
COX: cytochrome *c* oxidase
DPH: diphenylhexatriene
eIF2: eukaryotic initiation factor 2
ER: endoplasmic reticulum
Fura‐2/AM: 1‐[2‐(5‐carboxyoxazol‐2‐yl)‐6‐aminobenzofuran‐5‐oxy]‐2‐(2‐amino‐5‐methylphenoxy)‐ethane‐*N*,*N*,*N*′,*N*′‐tetraacetic acid pentaacetoxymethyl ester
GRP78: 78‐kDa glucose‐regulated protein
HMWC: high MW complex
IRE‐1α: inositol‐requiring transmembrane kinase/endoribonuclease 1α
MAM: mitochondria**‐**associated endoplasmic reticulum membrane
MDA: malondialdehyde
PAS: periodic acid–Schiff
PDA: pyrenedecanoic acid
PDI: protein disulfide isomerase
Penh: enhanced pause
PI3K: phosphatidylinositol 3‐kinase

**What is already known**

- PI3K‐δ and endoplasmic reticulum stress are involved in airway hyper‐responsiveness and refractory asthma.

**What this study adds**

- PI3K‐δ mediates changes in ER membrane fluidity and permeability in A.fumigatus‐induced allergic lung inflammation.
- Excessive ER–mitochondrial coupling is an essential mechanism for refractory asthma associated with ER stress.

**What is the clinical significance**

- PI3K‐δ and ER stress, along with ER–mitochondria interactions, underlie the development of refractory asthma.

## 1 INTRODUCTION

Fungi are increasingly recognised as the main cause of allergic asthma, one of the most common respiratory diseases that can lead to chronic airway inflammation, reversible airway obstruction, increased mucus production, and non‐specific airway hyper‐responsiveness (AHR; Leong & Huston, 2001). The fungus *Aspergillus fumigatus* is one of the most frequent stimuli inducing inflammation‐related airway AHR and remodelling (Hoselton, Samarasinghe, Seydel, & Schuh, 2010; Schuh & Hoselton, 2013). Furthermore, corticosteroids are not effective in the treatment of severe asthma associated with fungal sensitisation (Denning et al., 2009) and the alleviation of *A. fumigatus*‐induced allergic lung inflammation, including eosinophil‐dominant inflammatory cell infiltration into the lungs, AHR, and elevation of TH2‐dominant pro‐inflammatory cytokines (Felton, Lucas, Rossi, & Dransfield, 2014; Lee et al., 2016). This suggests that *A. fumigatus*‐induced lung inflammation represents a unique endotype of steroid‐resistant, eosinophil‐dominant, allergic lung inflammation (Felton et al., 2014; Lee et al., 2016). Exposure to the fungus activates https://www.guidetopharmacology.org/GRAC/ObjectDisplayForward?objectId=1754 signalling and consequently, https://www.guidetopharmacology.org/GRAC/FamilyDisplayForward?familyId=673 https://www.guidetopharmacology.org/GRAC/FamilyDisplayForward?familyId=285, and downstream NF‐κB pathways (Laird et al., 2009), leading to immune responses including the trafficking and activation of neutrophils and eosinophils (Kang et al., 2012; Puri et al., 2004). Thus, inhibition of https://www.guidetopharmacology.org/GRAC/ObjectDisplayForward?objectId=2155&familyId=673&familyType=ENZYME is a potential therapeutic strategy for refractory asthma.

PI3K activation is associated with high protein synthesis and folding load and affects the redox balance of protein folding organelles. Hyper‐loading of the endoplasmic reticulum (ER) also alters protein folding. To maintain its functional integrity, the ER must constantly balance the capacity of its protein chaperones with the load of newly synthesised unfolded proteins in the cell and disrupting this balance leads to the accumulation of unfolded or misfolded proteins in the ER (Hotamisligil, 2010). This process, known as ER stress, is associated with changes to the fluidity and permeability of the ER membrane (Kaplan, Racay, Lehotsky, & Mezesova, 1995; Pamplona, 2008) that result in the propagation of signals to nearby mitochondria (Malhotra & Kaufman, 2011; Marchi, Patergnani, & Pinton, 2014). The sites of physical contact between the ER and mitochondria—known as mitochondria‐associated endoplasmic reticulum membranes (MAMs)—are determinants of cell survival and death induced by inflammation signalling through the transfer of Ca^2+^, ROS, and other metabolites (Friedman et al., 2011; Rowland & Voeltz, 2012). However, the relevance of this interaction between organelles to inflammation‐related cellular dysfunction and metabolic homeostasis is not known.

In this study, we show that inhibiting PI3K‐δ suppresses fungus‐induced cytokine release and airway refractory asthma triggered by changes in ER membrane fluidity and permeability and abnormal MAM formation.

## 2 METHODS

### 2.1 Animals

All animal care and experimental procedures were approved by the Institutional Animal Care and Use Committee of Chonbuk National University (CBNU2017‐0039). All the animal studies complied with the principle of replacement, refinement, or reduction (the 3Rs). The animal studies are reported in compliance with the ARRIVE guidelines (Kilkenny et al., 2010) and with the recommendations made by the British Journal of Pharmacology. Female C57BL/6 mice aged 7–8 weeks and free of murine‐specific pathogens were obtained from Orient Bio Inc. Seoungnam, Korea. The mice were housed at 22 ± 1°C with 12‐hr light–dark cycles and fed with a regular chow diet and water ad libitum under standard conditions (specific pathogen free) with air filtration.

### 2.2 Experimental design

To establish the *A. fumigatus*‐induced allergic lung inflammation model, mice were treated with 10 μg of *A. fumigatus* crude antigen extract (Greer Laboratories, Cat# XPM3D3A4, Lenoir, NC, USA) in which the fungal material was inactivated and lyophilised and mixed with 0.2 ml of incomplete Freund's adjuvant (Sigma‐Aldrich, Cat# F5506‐6X) dissolved in normal saline. One‐half of this preparation was deposited in the peritoneal cavity, and the remainder was delivered subcutaneously. Two weeks later, the mice received 20 μg of *A. fumigatus* antigen dissolved in normal saline via the intranasal route and 4 days after intranasal challenge, the mice received 20 μg of *A. fumigatus* antigen dissolved in normal saline via the intratracheal route (Hogaboam et al., 2000). Non‐sensitised control mice were given normal saline alone via the same routes at the same time points and received the same number of conidia. Bronchoalveolar lavage (BAL) was performed in mice 48 hr after the last challenge with *A. fumigatus* (Figure S1).

A block randomisation technique was used to randomise the animals into groups of equal sample sizes at all time points. The optimum sample size and number of animals were determined by a power analysis. The experimental groups were designed as follows: 40 mice were divided randomly into four groups (*n* = 10): sham induction by the saline challenge (control group), asthma model with *A. fumigatus* induction only, *A. fumigatus* + https://www.guidetopharmacology.org/GRAC/LigandDisplayForward?ligandId=9376, and *A. fumigatus*+ 4‐phenylbutyric acid (4‐PBA). The investigators administering the treatments were blinded to group assignment. The numbers of mice used for each experimental group are shown in the figure legends. Data collection and evaluation of all experiments were performed without knowledge of the group identity. All the animal studies complied with the principle of replacement, refinement, or reduction (the 3Rs). A block randomisation technique was used to randomise the animals into groups of equal sample sizes at all time points. The investigators were blinded to the treatments. The animal studies are reported in compliance with the ARRIVE guidelines (Kilkenny et al., 2010) as per the recommendations of the British Journal of Pharmacology.

### 2.3 Drug administration

The selective PI3K‐δ inhibitor https://www.guidetopharmacology.org/GRAC/LigandDisplayForward?ligandId=9376 (1 mg kg^−1^ body weight day^−1^; Calbiochem, San Diego, CA, USA), chemical chaperone and the ER stress inhibitor 4‐PBA (Calbiochem; 80 mg kg^−1^ body weight day^−1^, diluted with PBS), or vehicle control (0.05% DMSO diluted in 0.9% NaCl solution) were administered by intratracheal injection twice to each animal, 24 hr before and after the last challenge with *A. fumigatus.*

### 2.4 Cell culture

BEAS‐2B (RRID:CVCL_0168) human bronchial epithelial cells were provided by Professor Hae‐Sim Park (Department of Allergy and Clinical Immunology, Ajou Research Institute for Innovation Medicine, Ajou University Medical Center, Suwon, South Korea). The cells were cultured and maintained at 80% confluence in RPMI‐1640 medium (Invitrogen, Carlsbad, CA, USA) with 10% FBS at 37°C in a humidified atmosphere of 5% carbon dioxide/95% air.

### 2.5 Analysis of bronchoalveolar lavage fluid (BALF) samples

BALF samples (1 ml) were obtained from each mouse and centrifuged (600× *g* for 3 min), and the supernatant was stored at −20°C for cytokine analysis. The cell pellets were pooled for determining total cell counts using a particle counter (Model Z1; Beckman‐Coulter, Miami, FL, USA) after lysing the erythrocytes (Zap‐Oglobin II; Beckman‐Coulter). Slides were loaded with cells, centrifuged (700× *g* for 3 min), and stained with Diff‐Quick (Baxter, Detroit, MI, USA). Lymphocytes, eosinophils, and neutrophils were counted under a light microscope.

### 2.6 Subcellular fractionation

Subcellular extractions (cytosol, nuclear, and ER) were performed as previously described (Kim et al., 2008). Briefly, lung tissue was resuspended in iso‐osmotic buffer (0.32‐M sucrose, 1‐mM MgCl_2_, 10‐mM Tris–HCl [pH 7.4]) and lysed by 20 passes with a Dounce homogeniser. The homogenate was centrifuged at 1,000× *g* for 10 min at 4°C to obtain the nuclear fraction (pellet). The supernatant was then centrifuged at 13,000× *g* for 30 min at 4°C, and the second supernatant was centrifuged at 100,000× *g* for 1 hr at 4°C using an SW32.1 rotor in an L8‐80M ultracentrifuge (Beckman‐Coulter) to obtain the cytosolic (supernatant) and ER (pellet) fractions. The fractions were stored at −80°C until use.

### 2.7 Immunohistochemistry and periodic acid–Schiff (PAS) staining

All antibody‐based procedures used in this study comply with the recommendations made by the *British Journal of Pharmacology.* Lung tissue samples were fixed in a 4% formalin solution for 24 hr, embedded in paraffin, and stained with antibodies or PAS reagent. Serial sections were cut at a thickness of 4 μm and subjected to antigen retrieval, blocked with 5% serum, and incubated overnight at 4°C with primary antibodies against mucin 5AC, 4‐HNE, GRP78, or CHOP. Immunoreactivity was visualised with 3,3′‐diaminobenzidine, and sections were counterstained with haematoxylin and eosin. Mucous cell metaplasia was quantified by analysing all the airways (large [conducting], medium [central], and small [distal]) present in representative lung sections. The number of airways containing PAS‐positive cells was counted; mucous cell metaplasia is shown as the percentage of airways with PAS‐positive cells and is expressed as the percentage of positive staining (malondialdehyde [MDA]) in the microscope field. Image analysis was performed using Metamorph Image Analysis software (Molecular Devices, Sunnyvale, CA, USA).

### 2.8 Western blotting, immunoprecipitation, and SDS‐PAGE

For western blotting, lung tissue samples were lysed in the following lysis buffer (10‐mM Tris [pH 7.4], 150‐mM NaCl, 1% Triton X‐100, 1% sodium deoxycholate, 0.1% SDS, 1‐mM phosphatase inhibitor cocktail, and 1‐mM protease inhibitor cocktail) for 30 min on ice. For immunoprecipitation, the lysate was first prepared in 50‐mM Tris–HCl, pH 8.0, containing 150‐mM NaCl, 0.015% phenylmethylsulphonyl fluoride, 1‐mM DTT, 1‐mM EDTA, 1% sodium deoxycholate, 1% Triton X‐100, and 1% SDS. The lysate was further diluted with the lysis buffer to achieve the desired volume and then incubated with anti‐PDIA6, anti‐p‐https://www.guidetopharmacology.org/GRAC/ObjectDisplayForward?objectId=2020, anti‐https://www.guidetopharmacology.org/GRAC/ObjectDisplayForward?objectId=1770, anti‐TXNIP, anti‐ASC, or anti‐https://www.guidetopharmacology.org/GRAC/ObjectDisplayForward?objectId=1617 antibody (1:100) overnight. Then, the lysates were incubated with 20 μl of protein A/G‐Sepharose (10% solution) for an additional 2 hr, and immunocomplexes were collected by centrifugation. The samples were then washed five times with 10‐mM Tris, pH 7.5 containing 0.1‐M NaCl and 1% Triton X‐100, and subsequently eluted in 50 μl of sample buffer. For SDS‐PAGE, equal amounts of protein were separated on a 10% SDS gel and transferred to a PVDF membrane using a mini‐transfer tank (Bio‐Rad, Hercules, CA, USA). The membrane was probed with primary antibodies; after incubation with secondary antibody, protein bands were detected with a chemiluminescence system. Protein expression levels were quantified and normalised based on the band intensity by using ImageJ 1.48v (http://imagej.nih.gov/ij, RRID:SCR_003070)..

### 2.9 Detection of cytokines in BALF

Cytokine levels in the BALF supernatant were quantified by enzyme immunoassay using commercial kits (BD Biosciences, Franklin Lakes, NJ, USA) according to the manufacturer's protocol.

### 2.10 Lipid peroxidation assay

Lipid peroxidation was assessed with a lipid hydroperoxide assay kit (Cayman Chemicals, Ann Arbor, MI, USA). Lung microsomes (1 mg) were homogenised in 1 ml of ice‐cold 2% SDS buffer. Sample homogenates and MDA standards were incubated with SDS and 0.8% thiobarbituric acid (20% acetic acid [pH 3.5]) in the presence of 0.8% butylated hydroxytoluene at 95°C for 1 hr. After cooling on ice and centrifuging at 1,000× *g* for 15 min, peroxidation levels in the supernatant were assessed by measuring the absorbance at 532 nm using a spectrophotometer.

### 2.11 GSH/GSSG ratio assay

Oxidative stress in the lung was evaluated using a https://www.guidetopharmacology.org/GRAC/LigandDisplayForward?ligandId=6737 assay kit (Cayman Chemicals) according to the manufacturer's instructions.

### 2.12 OxyBlot assay

The oxidative protein carbonylation assay was performed on lung tissue using an OxyBlot Protein Detection Kit (Millipore, Billerica, MA, USA) according to the manufacturer's instructions. The carbonyl groups in the protein side chains were derivatised to dinitrophenylhydrazine (DNP)‐hydrazone by reaction with 2,4‐DNP. The proteins were then separated by 10% SDS‐PAGE and transferred to a PVDF membrane. After incubation with an anti‐DNP antibody, the protein band was detected with a chemiluminescence system.

### 2.13 H_2_O_2_ measurement

H_2_O_2_ released from isolated ER was measured with the Amplex Red Hydrogen Peroxide/Peroxidase Assay Kit (Life Technologies, Darmstadt, Germany) according to the manufacturer's protocol in assay buffer composed of 115‐mM KCl, 10‐mM KH_2_PO_4_, 2‐mM MgCl_2_, 3‐mM HEPES, and 1‐mM EGTA (pH 7.2).

### 2.14 Membrane fluidity

ER membrane fluidity was evaluated with two different techniques (Ersoy, Maner‐Smith, Li, Alpertunga, & Cohen, 2018). The first measured changes in the fluorescence polarisation of diphenylhexatriene (DPH; Gibbons et al., 2013). Briefly, the ER microsomal fraction was resuspended in a KCl‐based buffer (150‐mM KCl, 10‐mM HEPES [pH 7.4], 2‐mM EGTA) and incubated with 10‐μM DPH at 45°C for 30 min. Endpoint fluorescence polarisation was measured with a Polarstar Omega plate reader (BMG Labtech, Ortenberg, Germany) at an excitation wavelength of 355 nm and parallel and perpendicular emission wavelengths of 440 ± 10 nm. The temperature was ramped from 25°C to 45°C in 2°C intervals. In the second approach, we measured the formation of pyrenedecanoic acid (PDA) excimers using the Membrane Fluidity Kit (MGT‐M0271; Axxora, Enzo Life Sciences, Farmingdale, NY, USA). ER microsomes were resuspended in PBS and incubated with 10‐μM PDA in the presence of 0.08% Pluronic F127 for 20 min at 25°C. After PDA incorporation into the membrane, the microsomes were washed three times with PBS to remove excess PDA and then resuspended in fresh PBS. Endpoint fluorescence was measured on a SpectraMax M5 microplate reader (Molecular Devices) at an excitation wavelength of 360 nm. PDA monomer and excimer emissions were detected at 400 and 470 nm, respectively. Microsome concentrations were titrated to obtain an even distribution of PDA molecules with an excimer‐to‐monomer ratio of 1 under basal conditions.

### 2.15 Measurement of intracellular Ca^2+^ ([Ca^2+^]_*i*_)

[Ca^2+^]_*i*_ was measured as previously described (Kim et al., 2008). The fluorescent calcium indicator 1‐[2‐(5‐carboxyoxazol‐2‐yl)‐6‐aminobenzofuran‐5‐oxy]‐2‐(2‐amino‐5‐methylphenoxy)‐ethane‐*N*,*N*,*N′*,*N′*‐tetraacetic acid pentaacetoxymethyl ester (Fura‐2/AM; Molecular Probes, Eugene, OR, USA) was used to detect changes in intracellular (cytosolic) free Ca^2+^. After Fura‐2/AM loading, cells were washed three times with isotonic KH buffer without Ca^2+^ (132‐mM NaCl, 5‐mM KCl, 10‐mM glucose, 10‐mM HEPES, and 1.05‐mM MgCl_2_) and then immediately treated with various agents including thapsigargin and ionomycin. Changes in [Ca^2+^]_*i*_ were determined as a ratio of 340‐/380‐nm excitation (512‐nm emission) using an integrated spectrofluorometer (Photon Technology International, Birmingham, NJ, USA). Ca^2+^ concentration was calculated using the equation [Ca^2+^]_*i*_ = *K*_D_ (*F*_380max_/*F*_380min_)(*R − R*_min_)/(*R*_max_ *− R*); a *K*_D_ value of 229 nM was assumed for the binding of Ca^2+^ to Fura‐2/AM. *R*_max_ and *R*_min_ were determined for each experimental group following the consecutive addition of 30‐μM Triton (*R*_max_) and 50‐mM EGTA (*R*_min_).

### 2.16 Data and statistical analysis

The data and statistical analysis comply with the recommendations of the *British Journal of Pharmacology* on experimental design and analysis in pharmacology (Curtis et al., 2018). The data are presented as the means ± *SD* of the measurements made with 10 mice in each group per experiment. The statistical analysis was performed using one‐way ANOVA followed by Tukey's post hoc test while comparing multiple independent groups. While comparing two different groups, unpaired *t* test was performed. Post hoc tests were used only if *F* achieved a *P*‐value of <.05 and there was no significant variance inhomogeneity. A *P*‐value of <.05 was considered statistically significant. The exact group size (*n*) for each experimental group/condition is provided, and “*n*” refers to the independent values, not replicates. For western blotting, membrane fluidity, and TXNIP localisation quantification, the results are expressed as fold difference as compared to the corresponding control values, and the control values were set as 1.0. For PDI activity, hydrogen peroxide assay, calcium content, and mitochondria ROS analysis, all values were normalised to the control group, and the *y*‐axis of the control group value was set to 100%. This normalisation process was used to minimise the background variations derived from the different experimental settings. The data were analysed using the software GraphPad Prism version 6.01 (La Jolla, CA, USA [RRID:SCR_002798]).

### 2.17 Nomenclature of targets and ligands

Key protein targets and ligands in this article are hyperlinked to corresponding entries in http://www.guidetopharmacology.org, the common portal for data from the IUPHAR/BPS Guide to PHARMACOLOGY (Harding et al., 2018), and are permanently archived in the Concise Guide to PHARMACOLOGY 2019/20 (Alexander, Fabbro et al., 2019a, 2019b; Alexander, Mathie et al., 2019).

### 2.18 Materials

Primary antibodies targeting the following proteins were used in this study: inositol‐requiring transmembrane kinase/endoribonuclease (IRE)‐1α (#3294, RRID:AB_823545) and CCAAT‐enhancer‐binding protein homologous protein (CHOP; #2895, RRID:AB_2089254; Cell Signaling Technology, Danvers, MA, USA); 78‐kDa glucose‐regulated protein (GRP78; https://www.scbt.com/scbt/ko/product/grp-78-antibody-76-e6?requestFrom=search, RRID:AB_627698) and β‐actin (https://www.scbt.com/scbt/ko/product/beta-actin-antibody-1?requestFrom=search, RRID:AB_630836; Santa Cruz Biotechnology, Santa Cruz, CA, USA); and phosphorylated p‐IRE‐1α (ab48287, RRID:AB_873899), 4‐hydroxynonenal (4‐HNE; ab48506, RRID:AB_722490), mucin 5AC (ab82423, RRID:AB_1861425), and monoclonal protein disulfide isomerase family A member (PDIA)6 (ab11432, RRID:AB_298038; Abcam, Cambridge, MA, USA). HRP‐conjugated secondary antibodies were purchased from Santa Cruz Biotechnology. IC87114 was from Yuhan Corporation (Seoul, Korea). *N*‐ethylmaleimide (E‐3876) and dihydroethidium (D7008) were from Sigma‐Aldrich (St. Louis, MO, USA).

## 3 RESULTS

### 3.1 PI3K‐δ is involved in airway inflammation, ER redox imbalance, and associated ROS accumulation in *A. fumigatus*‐induced allergic lung inflammation

**Figure 1:**
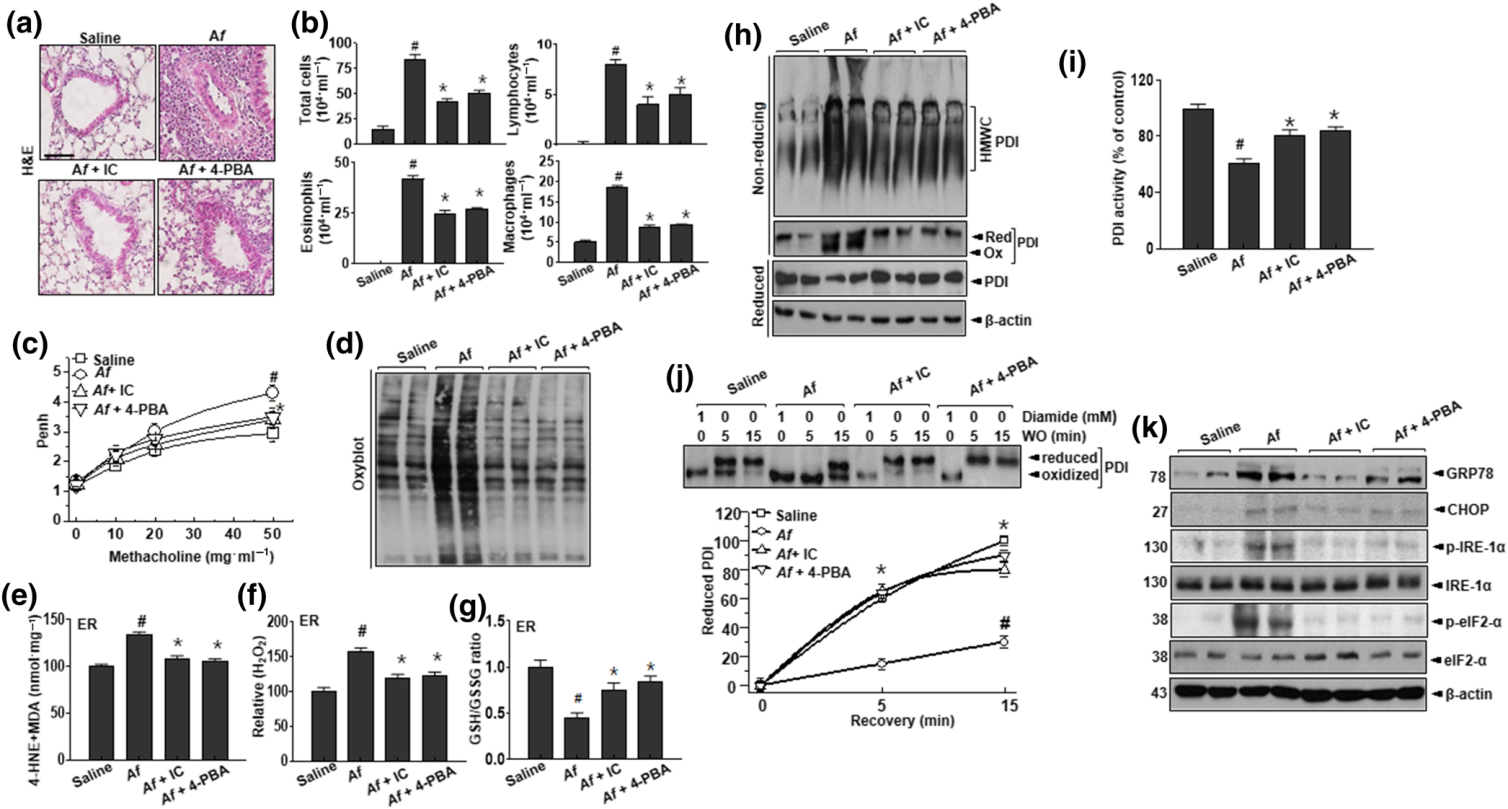
IC87114 and 4‐PBA attenuate airway inflammation and ROS generation in a mouse model of allergic lung inflammation induced by extracts of *A. fumigatus.* (a) Lung tissues and BALF cells obtained from *A. fumigatus‐* and saline‐treated (Af) mice and *A. fumigatus‐*challenged mice treated with 1 mg kg^−1^ IC87114 (Af + IC) or 80 mg kg^−1^ 4‐PBA (Af + 4‐PBA), were stained with haematoxylin and eosin. Scale bar 10 μm. (b) Total cells and distinct cellular components of BALF. (c) Airway reactivity in response to increasing doses of nebulised methacholine was assessed by whole‐body plethysmography. (d) Lysates from lung tissues were analysed for the presence of oxidised proteins by OxyBlot analysis. (e–g) MDA level (e), hydrogen peroxide level (f), and GSH/GSSG ratio (g) in the lung ER fraction. (h) HMWC formation was analysed using an anti‐PDI antibody. (i) PDI reductase activity in lung samples. Results are presented as the percentage of the PDI activity in control mice. (j) Lung lysates treated with 1‐mM diamide or left untreated for 15 min. After washout for the indicated time, the reduced and oxidised forms of PDI were detected by immunoblotting. (k) Immunoblot analysis of GRP78, CHOP, p‐IRE‐α, IRE‐α, and β‐actin expression in lung tissue; bands were quantified by densitometry and normalised to the level of β‐actin. Data are expressed as the mean ± *SD* (*n* = 10). ^#^ *P* < .05, significantly different from saline; *P < .05, significantly different from *A. fumigatus* alone; ANOVA

Previous studies have suggested that PI3K‐δ and ER stress are involved in airway inflammation and remodelling (Kim et al., 2018). To confirm the pathological role of PI3K‐δ and ER stress, we examined the effects of the PI3K‐δ inhibitor IC87114 and the chemical chaperone 4‐PBA that regulates ER stress in an *A. fumigatus*‐induced mouse model of airway inflammation. Histological analysis revealed greater infiltration of various types of inflammatory cells into the bronchioles in *A. fumigatus*‐exposed mice than in the control group (Figure 1a). As expected, *A. fumigatus*‐exposed mice treated with IC87114 or 4‐PBA showed a marked reduction in inflammatory cell infiltration. In particular, the eosinophil fraction was significantly increased in the BALF of *A. fumigatus*‐exposed mice, an effect that was mitigated by IC87114 or 4‐PBA administration (Figure 1b). Airway responses to methacholine were measured 1 hr before and 0, 4, and 8 hr after aspiration. Prior to aspiration, *A. fumigatus*‐exposed mice showed AHR, as shown by increased enhanced pause (Penh) values in response to methacholine. In contrast, IC87114 or 4‐PBA treatment reduced Penh values in *A. fumigatus*‐exposed mice (Figure 1c). PI3K activity and phosphorylation of the downstream target Akt were also inhibited by the administration of IC87114 or 4‐PBA (Figure S2A, B) whereas PAS staining and immunolabelling of mucin 5AC—a marker of mucus secretion—showed a decreased intensity (Figure S3). These results indicate that submucosal oedema and mucus hyper‐secretion are reduced by inhibiting PI3K‐δ and ER stress.

Given that oxidative stress is a hallmark of allergic lung inflammation (Lee et al., 2016), we analysed membrane lipid peroxidation, protein oxidation, and GSH redox status (GSH/GSSG balance) in our model of *A. fumigatus*‐induced asthma. The increase in protein oxidation observed in *A. fumigatus‐*challenged mice was reduced by treatment with https://www.guidetopharmacology.org/GRAC/LigandDisplayForward?ligandId=9376 or 4‐PBA (Figure 1d). As protein oxidation may be linked to ER‐associated ROS (Tu & Weissman, 2004), we assessed intra‐ER lipid peroxidation status with the 4‐HNE and MDA assays and by measuring H_2_O_2_ production and the GSH/GSSG ratio in mice exposed to *A. fumigatus*, with and without IC87114 or 4‐PBA treatment. The 4‐HNE and MDA levels in the ER fraction were reduced in the presence of IC87114 or 4‐PBA (Figure 1e), which also blocked the increase in intra‐ER H_2_O_2_ level in *A. fumigatus*‐exposed mice (Figure 1f). The balance between GSH and GSSG in the ER reflects ER protein oxidation status (Appenzeller‐Herzog, 2011); we found here that the GSH:GSSG ratio was decreased in *A. fumigatus*‐challenged mice and restored by IC87114 and 4‐PBA treatment (Figure 1g).

ER stress‐induced ROS generation impairs protein folding, resulting in the accumulation of insoluble multiprotein high MW complexes (HMWCs; Kenche, Baty, Vedagiri, Shapiro, & Blumental‐Perry, 2013) that disrupt ER functioning. *A. fumigatus‐*challenged mice exhibited increased HMWC formation, which was abolished by IC87114 or 4‐PBA (Figure 1h). Consistent with this finding, the decrease in PDI activity observed in *A. fumigatus‐*exposed mice was reversed by the administration of IC87114 or 4‐PBA (Figure 1i). To confirm the redox status of PDI, lung samples were exposed to the oxidative agent diamide (Appenzeller‐Herzog et al., 2010; Figure 1j). The oxidised form of PDI was readily converted to the reduced form of PDI under washout conditions with saline but persisted in *A. fumigatus*‐exposed samples. Interestingly, this was blocked by the presence of IC87114 or 4‐PBA in the washout condition. The expression of the ER chaperone GRP78, the phosphorylated protein kinase RNA‐like ER kinase, and the p‐IRE‐1α with its downstream effectors p‐eIF2 and CHOP—which constitute the branches of the adaptive unfolded protein response—were enhanced in *A. fumigatus*‐challenged mice compared with those in the saline control group. The elevated levels of ER stress markers in *A. fumigatus*‐challenged mice were also reduced by IC87114 and 4‐PBA, compared with vehicle‐treated mice (Figure 1k).

### 3.2 PI3K‐δ mediates changes in ER membrane fluidity and permeability in *A. fumigatus*‐induced allergic lung inflammation

**Figure 2:**
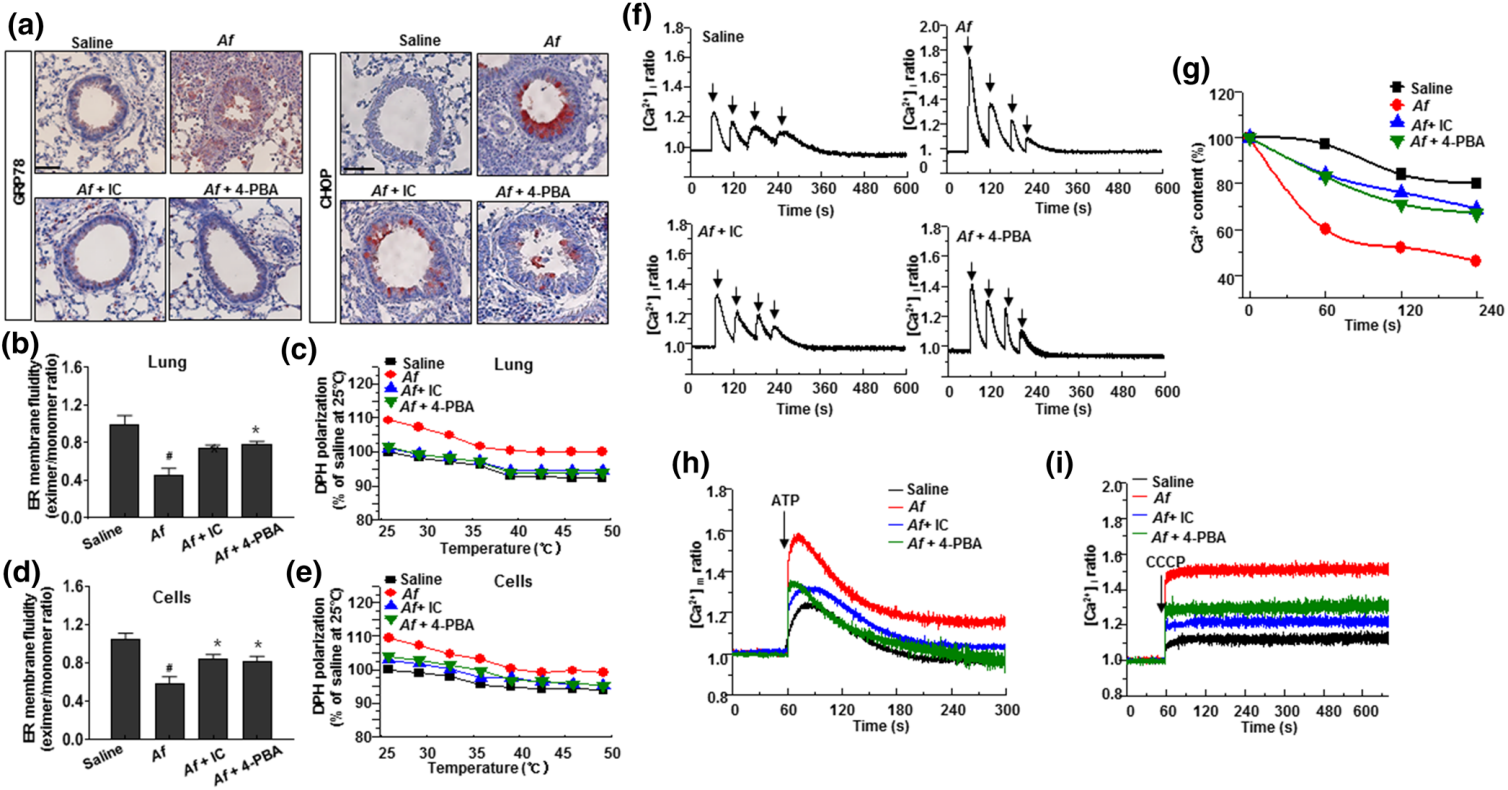
IC87114 and 4‐PBA restore ER membrane fluidity and calcium permeability in *Af‐*induced allergic lung inflammation. (a) Lung tissue samples were stained for GRP78 or CHOP. Scale bar 10 μm. (b–e) Lung tissue was obtained from *A. fumigatus‐*challenged and saline‐treated (Af) mice and *A. fumigatus‐*challenged mice treated with 1 mg kg^−1^ IC87114 (Af + IC) or 80 mg kg^−1^ 4‐PBA (Af + 4‐PBA) (b, c); and BEAS‐2B cells were treated with 0.1 mg ml^−1^ IC87114 or 5‐mM 4‐PBA with or without 100 μg ml^−1^ *A. fumigatus* for 24 hr prior to purification of ER fractions (d, e). ER membrane fluidity in lung tissues (b, c) and cells (d, e) were measured as the excimer‐to‐monomer ratio (b, d) or by the inverse correlation of DPH polarisation anisotropy, whereby decreased DPH polarisation indicates increased membrane fluidity (c, e). (f, g) Cells were treated with 0.1 mg ml^−1^ IC87114 or 5‐mM 4‐PBA with or without 100 μg ml^−1^ *A. fumigatus* for 24 hr before 1‐μM thapsigargin was added for 1 min; 10‐μM ionomycin was added either concomitantly with thapsigargin or at 1‐min intervals from 1 to 4 min. Ionomycin‐releasable Ca^2+^ (expressed as a percentage of the initial peak [Ca^2+^]_*i*_) is plotted as a function of time after thapsigargin addition. (h) [Ca^2+^]_*m*_ dynamics were measured following 100‐μM ATP stimulation. (i) Cytosolic Ca^2+^ was measured with 4‐μM Fura2‐AM after 1‐μM CCCP treatment. Data are expressed as the mean ± *SD* (*n* = 10). ^#^ *P* < .05, significantly different from saline; *P < .05, significantly different from *A. fumigatus* alone; ANOVA. CCCP, carbonyl cyanide m‐chlorophenyl hydrazine; i, intracellular; m, mitochondria

ER membrane properties such as fluidity and permeability can reflect ER stress status (Castuma & Brenner, 1983; Kaplan et al., 1995; Yang, Sheng, Sun, & Lee, 2011). In mice exposed to *A. fumigatus* and exhibiting an ER stress response (Figure 2a), we examined whether *A. fumigatus‐*induced allergic lung inflammation alters ER membrane fluidity, which could lead to the disruption of calcium homeostasis and increase in ER stress. In *A. fumigatus*‐exposed lung tissue and lung epithelial cells, ER membrane fluidity was decreased relative to the control, shown by the decrease in PDA excimer‐to‐monomer ratio (Figure 2b,c). Administration of IC87114 or 4‐PBA reversed this effect. These results were validated by using DPH polarisation anisotropy—which is based on the elimination of temperature‐dependent DPH polarisation (Stott et al., 2008)—to measure the membrane fluidity of purified ER fractions following treatment with IC87114 or 4‐PBA (Figure 2d,e).

ER membrane permeability, which is mechanistically linked to ER Ca^2+^ homeostasis, was evaluated by analysing the kinetics of thapsigargin‐induced Ca^2+^ depletion. Ionomycin was added after thapsigargin treatment to determine the amount of Ca^2+^ remaining in the ER by measuring cytosolic [Ca^2+^]_*i*_ in Fura‐2/AM‐loaded cells. In *A. fumigatus*‐pretreated BEAS‐2B lung epithelial cells, the amount of Ca^2+^ released in response to thapsigargin and ionomycin showed lower spikes in the presence of IC87114 or 4‐PBA than with saline treatment (Figure 2f,g). By adding ionomycin at different times after thapsigargin, we determined that the kinetics of Ca^2+^ release were markedly accelerated in *A. fumigatus‐*exposed cells; both IC87114 and 4‐PBA delayed Ca^2+^ release relative to the control group. In addition, we detected higher mitochondrial Ca^2+^ uptake after stimulating ER Ca^2+^ release with ATP in *A. fumigatus‐*challenged cells compared with the control group (Figure 2h). In the former, mitochondrial Ca^2+^ intake was reduced by IC87114 or 4‐PBA, while adding carbonyl cyanide m‐chlorophenyl hydrazone increased cytosolic Ca^2+^ content compared with the control (Figure 2i), an effect that was reversed by treatment with IC87114 or 4‐PBA.

### 3.3 PI3K‐δ is involved in the communication between ER and mitochondria in *A. fumigatus*‐induced allergic lung inflammation

**Figure 3:**
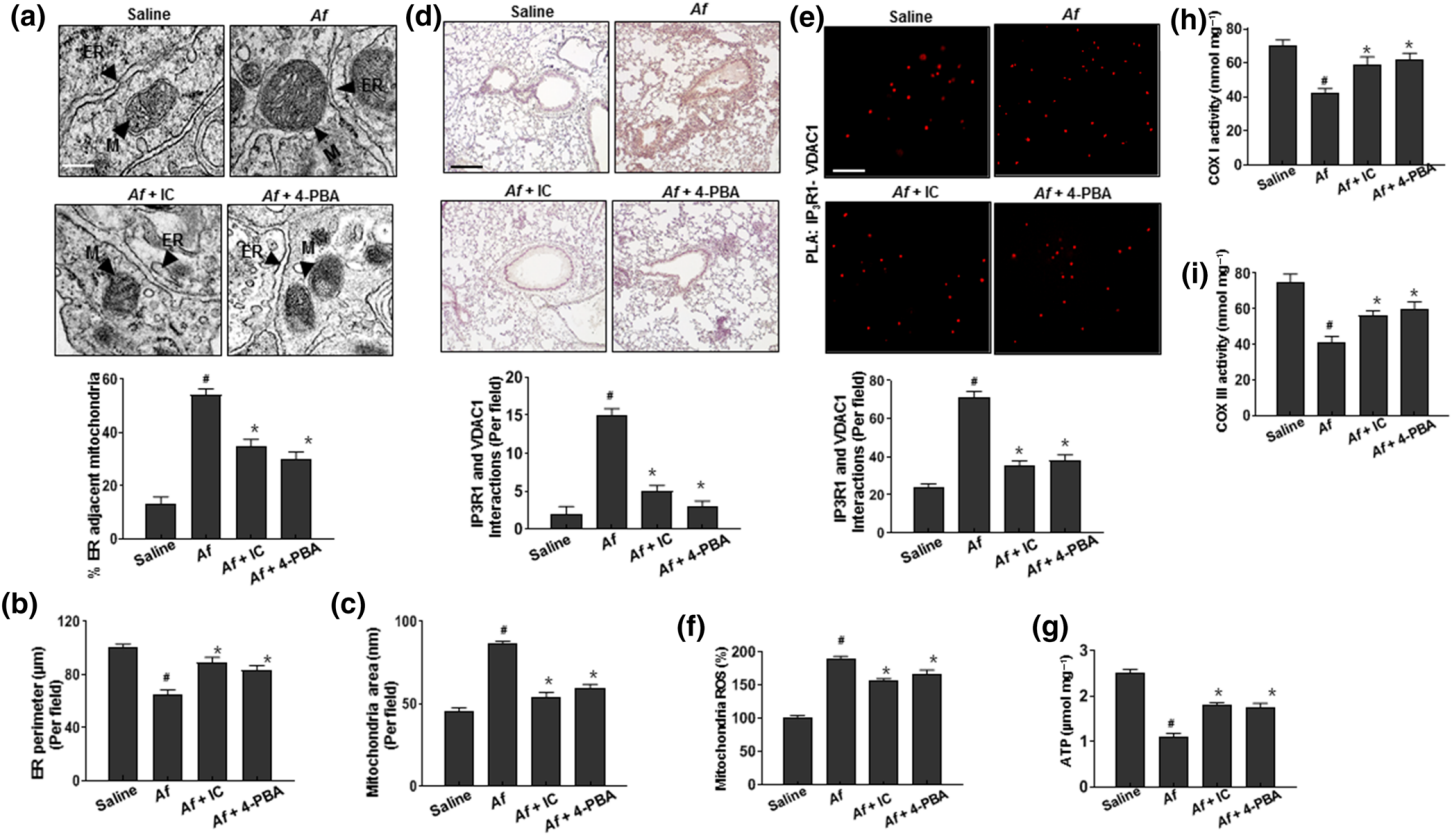
IC87114 and 4‐PBA decrease the number of ER–mitochondria contact sites in lung inflammation induced by extracts of *A. fumigatus* (a–e) Lung tissues were obtained from *A. fumigatus‐*challenged and saline‐treated (Af) mice and *A. fumigatus‐*challenged mice treated with 1 mg kg^−1^ IC87114 (Af + IC) or 80 mg kg^−1^ 4‐PBA (Af + 4‐PBA). Representative transmission electron micrographs (a), ER perimeter (b), mitochondria area (c), and results of the proximity ligation assay (PLA) assay with bright field (d) and fluorescence (e) images of lung sections are shown. Scale bar 10 μm. Quantitation of the number of positive PLA dots per field (https://www.guidetopharmacology.org/GRAC/FamilyDisplayForward?familyId=123#743 and VDAC1 interactions) are shown for indicated lung samples. (f–i) Mitochondrial ROS level (f), ATP level (g), and COX I (h) and COX III (i) activities. Data are expressed as the mean ± *SD* (*n* = 10). ^#^ *P* < .05, significantly different from saline; *P < .05, significantly different from *A. fumigatus* alone; ANOVA. IC, IC87114; IP3R1, IP_3_ receptor‐1; VDAC1, voltage‐dependent anion‐selective channel 1

As ER‐associated redox imbalance is amplified by juxtaposed mitochondria, the main source of cellular ROS (Yoboue, Sitia, & Simmen, 2018), we first confirmed the ER–mitochondria association under conditions of *A. fumigatus‐*induced ROS disturbance. In the lungs of *A. fumigatus* **‐**challenged mice, the ER membrane showed an aberrant morphology with an increased thickness and decreased perimeter, while the juxtaposed mitochondria appeared swollen, and the distance between the ER and mitochondria was smaller than normal; these phenotypes were reduced by treatment with IC87114 or 4‐PBA (Figure 3a–c). The interaction between the mitochondrial outer membrane protein, voltage‐dependent anion channel (VDAC)1, and the ER membrane‐associated channel protein https://www.guidetopharmacology.org/GRAC/FamilyDisplayForward?familyId=123#743 in the asthmatic condition was abolished in the presence of IC87114 or 4‐PBA, as determined with the proximity ligation assay (Figure 3d,e). We also analysed functional parameters of mitochondria including ROS levels, ATP, and cytochrome *c* oxidase (COX) I and COX III activity in *A. fumigatus‐*challenged mice (Figure 3f–i) and found that the exposure to *A. fumigatus* increased mitochondrial ROS and decreased ATP levels, while inhibiting COX I and COX III activities. These effects were reversed by IC87114 or 4‐PBA administration.

### 3.4 IC87114 and 4‐PBA attenuates the formation and activity of the NLRP3 inflammasome, induced by exposure to *A. fumigatus,* in airway inflammation

**Figure 4:**
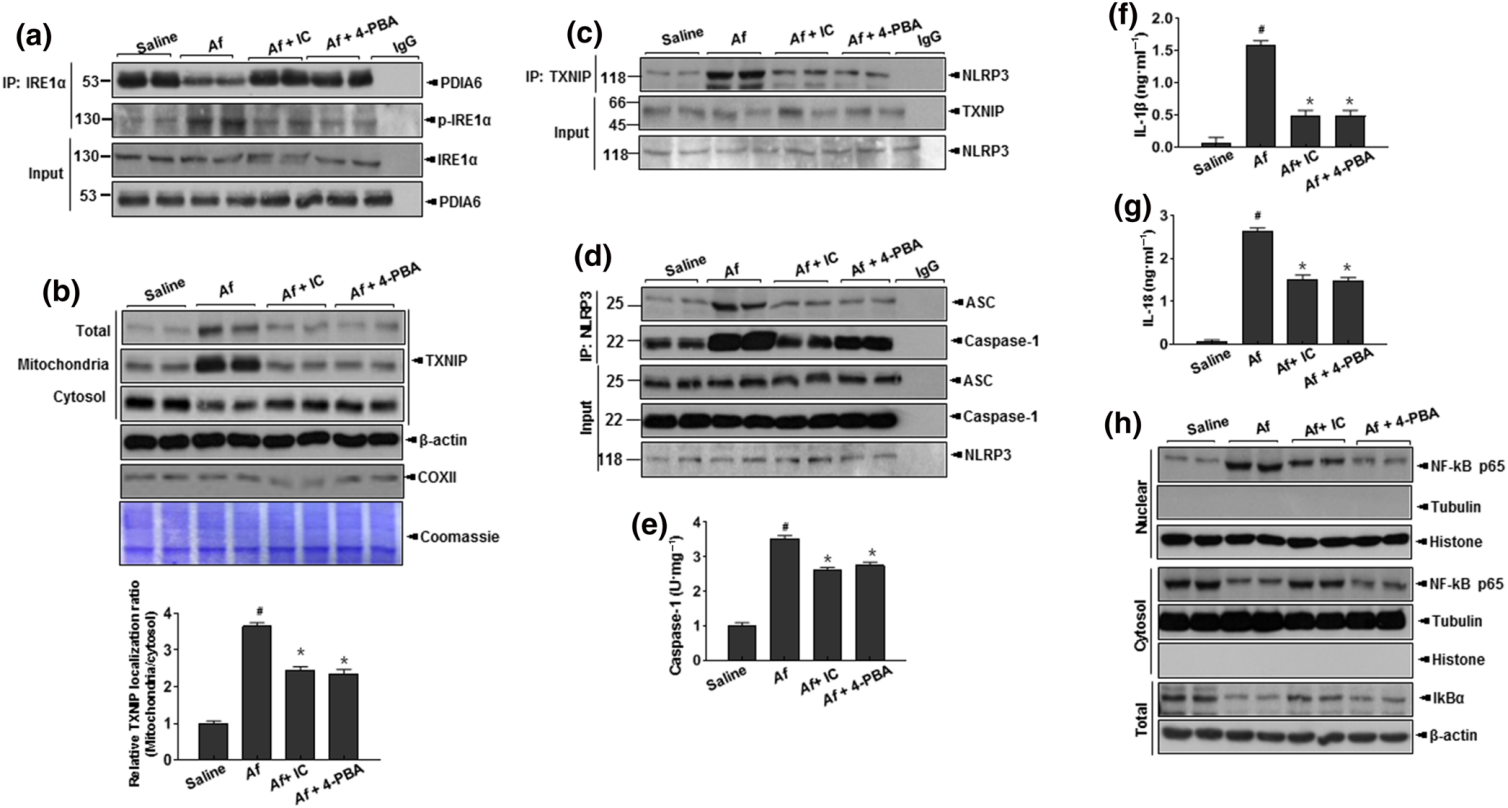
IC87114 and 4‐PBA alleviate *A. fumigatus‐*induced NLRP3 inflammasome activation of IRE1α. Lung tissues were obtained from *A. fumigatus‐*challenged, saline‐treated (Af) mice, and *A. fumigatus‐*challenged mice treated with 1 mg kg^−1^ IC87114 (Af + IC) or 80 mg kg^−1^ 4‐PBA (Af + 4‐PBA). (a) Lung tissue were immunoprecipitated and immunoblotted with anti‐IRE1α antibody and immunoblotted with anti‐PDIA6 antibody. (b) Subcellular localisation of TXNIP in membrane fractions of lung tissue was determined by immunoblotting and quantified. (c) Lung tissue lysates were immunoprecipitated with anti‐TXNIP antibody and immunoblotted with antibodies against TXNIP and NLRP3. (d) Lung tissue lysates were immunoprecipitated with anti‐NLRP3 antibodies and immunoblotted with antibodies against NLRP3, ASC, or caspase‐1. (e) Quantification of caspase‐1. ELISA analysis of released cytokines IL‐1β (f) and IL‐18 (g) in BALF. (h) Immunoblot analysis of nuclear NF‐κB p65/nuclear histone, cytosol p65/cytosolic tubulin, and IκB‐α/β‐actin; bands were quantified by densitometry and normalised to histone (nuclear), tubulin (cytosol), and β‐actin (total) as the control. Data are expressed as the mean ± *SD* (*n* = 10). ^#^ *P* < .05, significantly different from saline; *P < .05, significantly different from *A. fumigatus* alone; ANOVA. ER, endoplasmic reticulum; IC, IC87114; TXNIP, thioredoxin interacting protein; NLRP3, NOD‐like receptor protein

The kinase IRE1α is the main ER stress sensor, and its RNAse activity is linked to ER–mitochondria communication through contact between the organelles ( Carreras‐Sureda et al., 2019). Upon the activation of IRE1α, the binding proteins GRP78 and PDIA6 dissociate from it. As expected, PDIA6 dissociated from IRE1α, yielding p‐IRE1α in the *A. fumigatus*‐challenged mice, but it was stably bound to IRE1α in the presence of IC87114 or 4‐PBA (Figure 4a). In the *A. fumigatus*‐challenged mice, the activation of IRE1α increased the mitochondrial enrichment of thioredoxin interacting protein (TXNIP; Figure 4b), which shuttles to mitochondria and binds thioredoxin‐2, thereby increasing the concentration of mitochondrial ROS (Lerner et al., 2012). The mitochondrial expression of TXNIP was significantly decreased by the administration of IC87114 or 4‐PBA. The interaction between TXNIP and NLRP3—the main mechanism for inflammasome formation and the resultant NF‐κB activation during allergic lung inflammation—was clearly observed in the *A. fumigatus*‐challenged condition, whereas administration of IC87114 or 4‐PBA suppressed the association between TXNIP and NLRP3 (Figure 4c).

Allergic lung inflammation evokes a clear pro‐inflammatory shift in cytokine expression through the formation of the inflammasome (Chen & Nunez, 2010; Rincon & Irvin, 2012). We confirmed the association of NLRP3**,** ASC, and caspase‐1 in *A. fumigatus*‐exposed mice (Figure 4d) and showed that the complex was dissociated by the administration of IC87114 or 4‐PBA. The activation of https://www.guidetopharmacology.org/GRAC/ObjectDisplayForward?objectId=1617 (Figure 4e) and subsequent release of mature https://www.guidetopharmacology.org/GRAC/LigandDisplayForward?ligandId=4974 (Figure 4f) and https://www.guidetopharmacology.org/GRAC/LigandDisplayForward?ligandId=4983 (Figure 4g) were increased in the *A. fumigatus*‐induced mice, whereas levels of those cytokines were drastically reduced by IC87114. Consistent with those data, the levels of https://www.guidetopharmacology.org/GRAC/LigandDisplayForward?ligandId=4996, https://www.guidetopharmacology.org/GRAC/LigandDisplayForward?ligandId=4997, https://www.guidetopharmacology.org/GRAC/LigandDisplayForward?ligandId=4980, and https://www.guidetopharmacology.org/GRAC/FamilyDisplayForward?familyId=313 were decreased by the administration of IC87114 or 4‐PBA (Figure S5A–D). Subcellular fractionation showed that the nuclear translocation of NF‐κB and the degradation of cytosolic IκB‐α were significantly inhibited by the administration of IC87114 or 4‐PBA (Figure 4h).

Under conditions of physical contact between the ER and mitochondria, permeable ions and metabolites are transferred from one to the other in both directions. The mitochondrial outer membrane is permeable to both ROS and calcium leaked from the ER through VDAC1, which is linked to mitochondrial metabolic activity and ultimately required for ROS production (Colombini, 2004). As expected, VDAC1 was highly expressed in *A. fumigatus*‐induced asthma, and its expression was controlled by treatment with IC87114 or 4‐PBA (Figure S6A). In cells with VDAC1 knockdown, the associations of ASC (apoptosis**‐**associated speck**‐**like protein containing a CARD) and activated caspase‐1 with NLRP3 were markedly blocked (Figure S6B), indicating that in the inflamed condition, a substantial proportion of the inflammasome is associated with MAMs. Furthermore, NLRP3 checks and modulates mitochondrial ROS and the subsequently linked NF‐κB activity and that association is negatively controlled by treatment with either a PI3K‐δ inhibitor or a chemical chaperone.

## 4 DISCUSSION

The results of this study demonstrate that inhibiting PI3K‐δ or ER stress can mitigate airway inflammation and remodelling in an *A. fumigatus*‐induced mouse model of severe asthma characterised by ER stress, changes in ER membrane fluidity and permeability, close contact between the ER and mitochondria, and amplified Ca^2+^ and ROS‐induced signalling. In airway remodelling, the PI3K‐δ–ER stress axis provides a physical link between ER and mitochondria and mitochondrial ROS accumulation, which leads to NF‐κB activation.

In *A. fumigatus*‐induced airway remodelling, ER redox imbalance and the associated impairment of protein folding capacity are related to PI3K‐δ activation and ER stress (Dai, Chen, & Li, 2009; Kadowaki & Nishitoh, 2013). Our data revealed intra‐ER folding in the hyper‐loaded state—including ER hyperoxidation and PDI‐associated oxidised protein accumulation—and a decreased ratio of reduced to oxidised GSH, which undermines the capacity of the ER for thiol oxidation (Figure 1). An increased protein load that includes inflammatory cytokines is linked to translational stress involving eIF2α and https://www.guidetopharmacology.org/GRAC/ObjectDisplayForward?objectId=2030 (Shin et al., 2014; Zhang & Kaufman, 2008) and can induce other ER stress responses involving IRE‐1α and downstream signalling (Ron, 2002). In addition, dexamethasone failed to alleviate *A. fumigatus* ‐induced airway remodelling and eosinophil‐dominant inflammatory cell infiltration in the lungs and elevation of Th2‐cytokines suggesting that steroid‐resistant *A. fumigatus*‐induced pulmonary dysfunction could be successfully controlled by the regulation of the PI3K–ER stress axis (Figure S7). The combination therapy, IC87114 and 4‐PBA, had no additive effects against the representative endotypes of pulmonary inflammation. Furthermore, in the presence of an airway inflammatory condition, IC87114 regulates the downstream ER stress signalling (IRE‐1α and PERK; Figures 1k and 2a) and 4‐PBA prevents Akt activation, as observed with the PI3K inhibitor IC87114 (Figure S2A, B). This indicates the mutual crosstalk signalling between the PI3K and ER stress. Consistent with our findings, other studies have also described the crosstalk signalling network, “PI3K–ER stress axis,” in different metabolic/inflammatory conditions including diabetes, cancer and asthma (Hsu et al., 2017; Qin, Wang, Tao, & Wang, 2010).

The *A. fumigatus*‐induced ER stress resulted in ER membrane peroxidation both in vivo and in vitro, which decreased ER membrane fluidity, increased its permeability, and altered the capacity of ER vesicles to sequester Ca^2+^ ions (Figure 2). Previous reports indicate that ER membrane‐associated oxidative stress induces crosslinking via formation of disulfide bridges from two intermolecular thiol (─SH) groups in proteins to lipid membrane rafts as well as formation of adducts with MDA, a typical lipid peroxidation product. This can lead to peroxidative changes in polyunsaturated fatty acid content and membrane dysfunction caused by increased tissue permeability through the loss of membrane fluidity, which is implicated in lung inflammation (Chen & Yu, 1994). Our study also showed that ER membrane peroxidation decreased ER membrane fluidity in lung tissue and pulmonary epithelial cells, which could underlie the observed imbalance in ER redox state and Ca^2+^ sequestration. Thus, activation of the PI3K‐δ–ER stress signalling axis is associated with altered ER membrane fluidity and permeability and may serve as the pathological basis for fungus‐induced airway remodelling.

The functional alteration of ER membranes affects the physical state of ER and mitochondria leading to MAM formation. Cross‐communication between these two organelles might be due to the close physical proximity of the two organelles (Figure 3a,e). In the presence of severe inflammation and damage manifested by alteration of ER membrane fluidity and permeability, the MAM, a platform for crosstalk between the ER and mitochondria, might be easily formed, promoting increased production of ROS and release of mitochondrial DAMPs into the cytosol (Thoudam, Jeon, Ha, & Lee, 2016). In the process of inflammasome formation, NLRP3 binds to the signalling adaptor TXNIP, whose translation is controlled by the activated IRE1α (Figure 4), an ER stress signalling protein, that also controls the opening of the mitochondrial Ca^2+^ intake pore VDAC. Mitochondrial electron transfer chain coupling and uncoupling, a key signalling pathway for mitochondrial ROS, is greatly affected by mitochondrial Ca^2+^, an important regulator of mitochondrial depolarisation linked to inflammation (Giorgi et al., 2012; Tait & Green, 2012). VDAC expression was increased at the mitochondrial membrane‐enriched sites of the ER in the *A. fumigatus*‐induced recruitment of inflammatory cells and NF‐κB signalling (Figure S6A). Silencing VDAC with siRNA emphasised the critical role of mitochondrial Ca^2+^ in NLRP3, caspase‐1, and mitochondrial damage in the active state of the PI3K‐δ–ER stress axis (Figure S6B). Consistent with our results, mitochondrial signalling pathways that communicate with the other organelles has been demonstrated in asthma‐associated inflammations (Arruda et al., 2014; Thoudam et al., 2016) and is thought to contribute to Ca^2+^ and lipid transfer and ROS amplification in the inflammatory NF‐κB signalling (Csordas & Hajnoczky, 2009; Gorlach, Bertram, Hudecova, & Krizanova, 2015). In this study, the PI3K–ER stress axis has been strongly suggested to be a NF‐κB signalling pathway for the amplification of inflammation, where ER–mitochondria connection‐based mitochondrial characteristics were functionally changed through ER membrane fluidity and alteration in the permeability.

In conclusion, our results suggest that PI3K‐δ and ER stress along with ER–mitochondria interactions are involved in the mechanism behind refractory asthma and inflammation, which is challenging to study. Further studies should consider testing components of this mechanism as therapeutic targets against pulmonary diseases involving inflammation and immunity.

## AUTHOR CONTRIBUTIONS

H.Y.L. performed the experiments and data analysis. H.Y.L. and G.H.L. carried out data statistical analysis. H.Y.L. and G.H.L. performed animal studies. H.R.K. read and corrected the manuscript. H.J.C. conceived, supervised, and funded the work. All authors reviewed the manuscript.

## CONFLICT OF INTEREST

The authors declare no conflicts of interest.

## DECLARATION OF TRANSPARENCY AND SCIENTIFIC RIGOUR

This Declaration acknowledges that this paper adheres to the principles for transparent reporting and scientific rigour of preclinical research as stated in the *BJP* guidelines for https://bpspubs.onlinelibrary.wiley.com/doi/abs/10.1111/bph.14207, https://bpspubs.onlinelibrary.wiley.com/doi/abs/10.1111/bph.14208 and https://bpspubs.onlinelibrary.wiley.com/doi/abs/10.1111/bph.14206, and as recommended by funding agencies, publishers and other organizations engaged with supporting research.

## Supporting information


**Figure S1.** Experimental design and time course of bronchoalveolar lavage inflammatory cell infiltration. Mice received an intraperitoneal and subcutaneous injection of soluble *A. fumigatus (Af)* antigens dissolved in incomplete Freund's adjuvant. Two weeks after systemic sensitization, each mouse then received a intranasal challenge with *Af* antigen to localize the allergic responsiveness to the airways. One week after the intranasal challenge, each mouse then received 5.0×10^6^ *Af* conidia suspended in 50 μl via the intratracheal route. Nonsensitized mice received normal saline alone via the same routes and over the same time periods, and received the same number of conidia. A selective p110‐δ inhibitor IC87114 (1 mg/kg body weight/day, Calbiochem, San Diego, CA, USA), chemical chaperone, 4‐phenylbutyricacid (4‐PBA, Calbiochem; 80 mg/kg body weight/day, diluted with phosphate‐buffered saline), or vehicle control (0.05% dimethyl sulfoxide [DMSO] diluted with 0.9% NaCl) were administered twice by intratracheal injection to each animal, 24 h before and after the last challenge with *Af*. *Af, Aspergillus fumigatus*; IC, IC87114; 4‐PBA, 4‐phenylbutyric acid
**Figure S2**. IC87114 and 4‐PBA attenuates airway inflammation in *Aspergillus fumigatus (Af)‐*induced allergic lung inflammation. Lung tissues were obtained from *Af‐*challenged mice, saline‐treated mice, and *Af‐*challenged mice treated with 1 mg/kg IC87114 or 80 mg/kg 4‐PBA. (A) Immunoblotting and densitometric analyses (lower) were performed with anti‐p‐AKT or AKT antibody. (B) PI3K activity was measured as described in Methods. Data are expressed as the mean ± SD and were analysed by ANOVA (n=10). (^#^ *p* < 0.05 versus saline; ^*^ *p* < 0.05 versus *Af*). *Af, Aspergillus fumigatus*; IC, IC87114; 4‐PBA, 4‐phenylbutyric acid
**Figure S3**. IC87114 and 4‐PBA attenuate airway inflammation in *Aspergillus fumigatus (Af)‐*induced allergic lung inflammation model. (A) Lung tissues and BALF cells obtained from *Af‐*treated mice, saline‐treated mice, and *Af‐*challenged mice treated with 1 mg/kg IC87114 or 80 mg/kg 4‐PBA were stained with PAS (upper) and anti‐mucin5AC antibody (lower) showing the quantitation of positive cells. Data are expressed as the mean ± SD and were analysed by ANOVA (n = 10). (^#^ *p* < 0.05 versus saline; ^*^ *p* < 0.05 versus *Af*). *Af*, *Aspergillus fumigatus*; IC, IC87114; 4‐PBA, 4‐ phenylbutyric acid; PAS, periodic acid‐Schiff.
**Figure S4.** IC87114 and 4‐PBA alleviates *Aspergillus fumigatus‐*induced RIG‐1 signaling by controlling RIDD activity. (A) mRNA levels of known IRE1‐RIDD target genes *Blos1*, *Pdqfrb,* and *Pmp2*. (B) Lung lysates were subjected to immunoblotting and immunoprecipitation with antibodies against MAVS or RIG‐I. Data are expressed as the mean ± SD and were analysed by ANOVA (n = 10). (^#^ *p* < 0.05 vs. saline; ^*^ *p* < 0.05 vs. *Af*). *Af, Aspergillus fumigatus*; IC, IC87114; 4‐PBA, 4‐phenylbutyric acid
**Figure S5**. IC87114 and 4‐PBA reduce inflammatory signaling in lungs and BAL fluids of *Aspergillus fumigatus (Af)‐*induced allergic lung inflammation. BAL fluids were collected and analyzed by ELISA for the cytokines IL‐4 (A), IL‐5 (B), IL‐13 (C), and IL‐17 (D). Data are expressed as the mean ± SD and were analysed by ANOVA (n = 10). (^#^ *p* < 0.05 vs. saline; ^*^ *p* < 0.05 vs. *Af*). *Af, Aspergillus fumigatus*; IC, IC87114; 4‐PBA, 4‐phenylbutyric acid
**Figure S6**. VDAC is essential for NLRP3 inflammasome activation. Lung tissues were obtained from *Af‐*challenged mice, saline‐treated mice, and *Af‐*challenged mice treated with 1 mg/kg IC87114 or 80 mg/kg 4‐PBA. (A) Immunoblot analysis of VDAC1 (outer mitochondrial membrane marker), IP3R1 (ER marker), Sig1R (mitochondria‐associated endoplasmic reticulum membranes marker), and PDI (*ER marker*) in MAM fractions of lung tissue. (B) BEAS‐2B cells transiently transfected with non‐specific siRNA or stably expressing VDAC1‐specific siRNA were subjected to immunoblotting with anti‐VDAC1 or β‐actin antibody. BEAS‐2B cells were treated with 0.1 mg/ml or 5 mM 4‐PBA with or without 100 μg/mL *Af* for 24 h. BEAS‐2B cell expressing siRNA against VDAC1 were immunoprecipitated with anti‐NLRP3 antibody and immunoblotted with antibody against NLRP3, ASC, and caspase‐1. MAM, mitochondria associated ER membranes; *Af*, *Aspergillus fumigatus*; IC, IC87114; 4‐PBA, 4‐phenylbutyric acid
**Figure S7**. Dexamethasone does not affect inflammation in *Af‐*induced allergic lung inflammation model. (A) The lung tissues and BALF cells obtained from *Af‐* and saline‐treated mice and *Af‐*challenged mice, treated with 1 mg/kg IC87114, 80 mg/kg 4‐PBA, 1 mg/kg IC87114 + 80 mg/kg 4‐ PBA, or 1 mg/kg dexamethasone, were stained with haematoxylin and eosin. (B) The entire cells and the distinct cellular components of BALF. (C) BAL fluids were collected and the levels of the cytokines (IL‐4, IL‐5, IL‐13, and IL‐17) were estimated by ELISA. Data are expressed as the mean ± SD and were analysed by ANOVA (n = 10).. ^#^ *p* < 0.05 vs. saline; ^*^ *p* < 0.05 vs. *Af*. *Af, Aspergillus fumigatus*; IC, IC87114; 4‐PBA, 4‐phenylbutyric acid
