# Supplementary material for: Phosphatidylinositol 3‐kinase‐δ controls endoplasmic reticulum membrane fluidity and permeability in fungus‐induced allergic inflammation in mice
Source: Br J Pharmacol. 2020 Jan 27;177(7):1556–67. doi: 10.1111/bph.14917 (PMC7060358; doi:10.1111/bph.14917)
Supplement: Supplementary file 1 — Figure S1. Experimental design and time course of bronchoalveolar lavage inflammatory cell infiltration. Mice received an intraperitoneal and subcutaneous injection of soluble A. fumigatus (Af) antigens dissolved in incomplete Freund's adjuvant. Two weeks after systemic sensitization, each mouse then received a intranasal challenge with Af antigen to localize the allergic responsiveness to the airways. One week after the intranasal challenge, each mouse then received 5.0×10^6^ Af conidia suspended in 50 μl via the intratracheal route. Nonsensitized mice received normal saline alone via the same routes and over the same time periods, and received the same number of conidia. A selective p110‐δ inhibitor IC87114 (1 mg/kg body weight/day, Calbiochem, San Diego, CA, USA), chemical chaperone, 4‐phenylbutyricacid (4‐PBA, Calbiochem; 80 mg/kg body weight/day, diluted with phosphate‐buffered saline), or vehicle control (0.05% dimethyl sulfoxide [DMSO] diluted with 0.9% NaCl) were administered twice by intratracheal injection to each animal, 24 h before and after the last challenge with Af. Af, Aspergillus fumigatus; IC, IC87114; 4‐PBA, 4‐phenylbutyric acid Figure S2. IC87114 and 4‐PBA attenuates airway inflammation in Aspergillus fumigatus (Af)‐induced allergic lung inflammation. Lung tissues were obtained from Af‐challenged mice, saline‐treated mice, and Af‐challenged mice treated with 1 mg/kg IC87114 or 80 mg/kg 4‐PBA. (A) Immunoblotting and densitometric analyses (lower) were performed with anti‐p‐AKT or AKT antibody. (B) PI3K activity was measured as described in Methods. Data are expressed as the mean ± SD and were analysed by ANOVA (n=10). (# p < 0.05 versus saline; * p < 0.05 versus Af). Af, Aspergillus fumigatus; IC, IC87114; 4‐PBA, 4‐phenylbutyric acid Figure S3. IC87114 and 4‐PBA attenuate airway inflammation in Aspergillus fumigatus (Af)‐induced allergic lung inflammation model. (A) Lung tissues and BALF cells obtained from Af‐treated mice, saline‐treated mice, and [file BPH-177-1556-s001.pdf]

PI3K-δ controls endoplasmic reticulum membrane fluidity and permeability in fungus-induced allergic inflammation

Hwa-Young Lee[1], Geum-Hwa Lee[1], Hyung-Ryong Kim[2], Yong-Chul Kim[3], Han-Jung Chae[1¶]

[1]Department of Pharmacology and Institute of New Drug Development, Chonbuk National University Medical School, Jeonju, Chonbuk, 561-180, Republic of Korea; [2]Daegu Gyeonbuk Institute of Science & Technology (DGIST) graduate school, Daegu, Republic of Korea, [3]Department of Internal Medicine, Chonbuk National University Medical School, Jeonju, Chonbuk 561-180, Republic of Korea

[¶]Corresponding authors: Han-Jung Chae, PhD, Department of Pharmacology and Institute of New Drug Development, Chonbuk National University Medical School, Jeonju, Chonbuk, 561-180, South Korea. Tel: 82-63-270-3092, Fax: 82-63-275-2855; E-mail: hjchae@jbnu.ac.kr

Running title

The Communication between ER and Mitochondria

Supplementary Figure 1

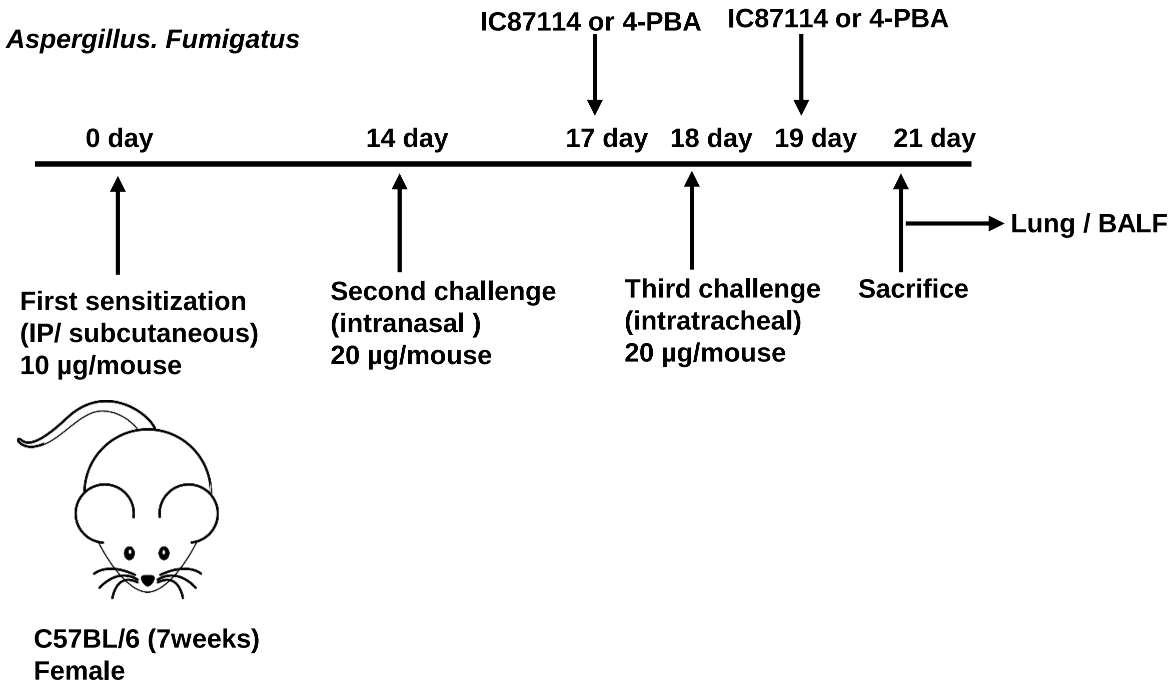

Supplementary Figure 1. Experimental design and time course of bronchoalveolar lavage inflammatory cell infiltration. Mice received an intraperitoneal and subcutaneous injection of soluble *A. fumigatus (Af)* antigens dissolved in incomplete Freund′s adjuvant. Two weeks after systemic sensitization, each mouse then received a intranasal challenge with *Af* antigen to localize the allergic responsiveness to the airways. One week after the intranasal challenge, each mouse then received 5.0 × 10^6 *Af* conidia suspended in 50 μl via the intratracheal route. Nonsensitized mice received normal saline alone via the same routes and over the same time periods, and received the same number of conidia. A selective p110-δ inhibitor IC87114 (1 mg/kg body weight/day, Calbiochem, San Diego, CA, USA), chemical chaperone, 4-phenylbutyric acid (4-PBA, Calbiochem; 80 mg/kg body weight/day, diluted with phosphate-buffered saline), or vehicle control (0.05% dimethyl sulfoxide [DMSO] diluted with 0.9% NaCl) were administered twice by intratracheal injection to each animal, 24 h before and after the last challenge with *Af*. *Af*, *Aspergillus fumigatus*; IC, IC87114; 4-PBA, 4-phenylbutyric acid

Supplementary Figure 2

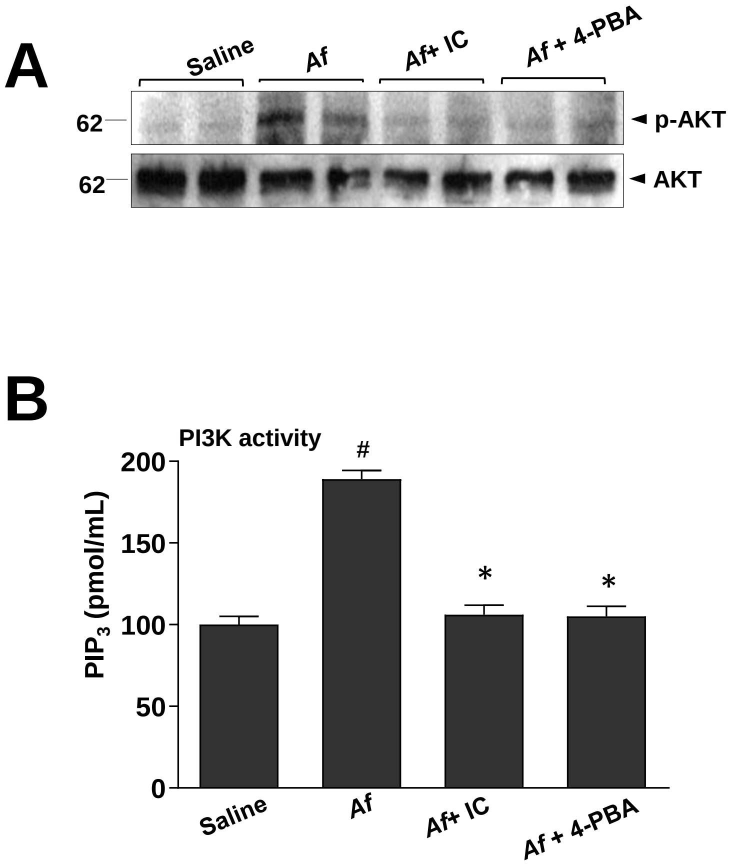

Supplementary Figure 2. IC87114 and 4-PBA attenuates airway inflammation in *Aspergillus fumigatus (Af)*-induced allergic lung inflammation. Lung tissues were obtained from *Af*-challenged mice, saline-treated mice, and *Af*-challenged mice treated with 1 mg/kg IC87114 or 80 mg/kg 4-PBA. (A) Immunoblotting and densitometric analyses (lower) were performed with anti-p-AKT or AKT antibody. (B) PI3K activity was measured as described in Methods. Data are expressed as the mean ± SD and were analysed by ANOVA (n=10). ($^{\#}p < 0.05$ versus saline; $^{*}p < 0.05$ versus *Af*). *Af, Aspergillus fumigatus*; IC, IC87114; 4-PBA, 4-phenylbutyric acid

Supplementary Figure 3

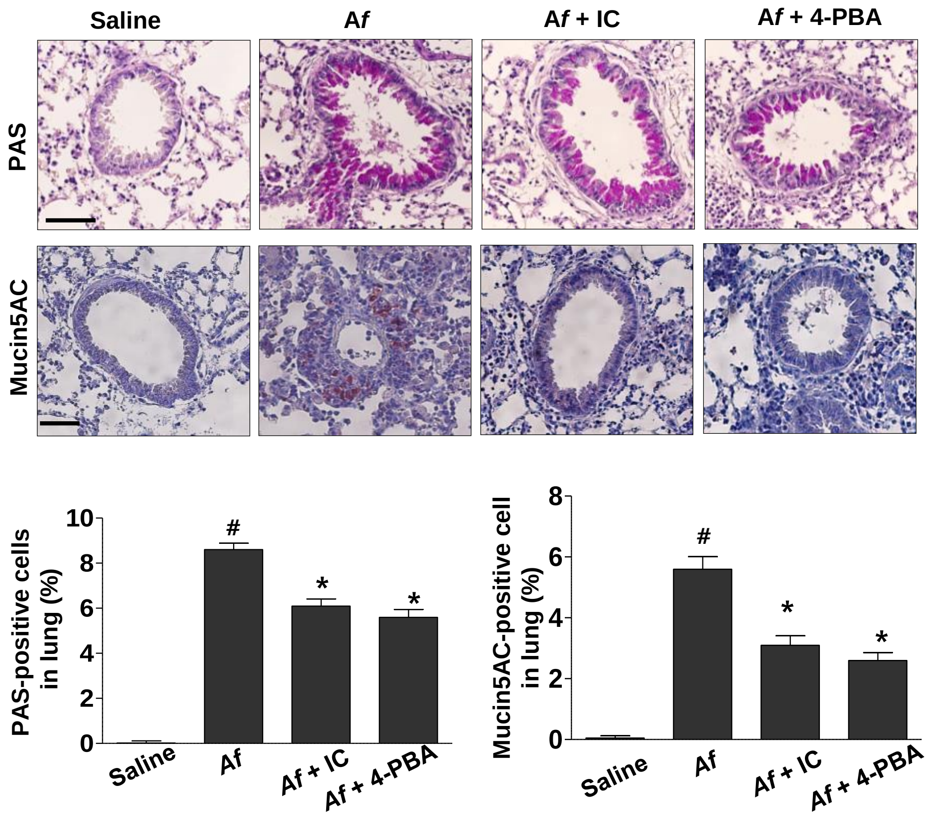

Supplementary Figure 3. IC87114 and 4-PBA attenuate airway inflammation in *Aspergillus fumigatus (Af)*-induced allergic lung inflammation model. (A) Lung tissues and BALF cells obtained from *Af*-treated mice, saline-treated mice, and *Af*-challenged mice treated with 1 mg/kg IC87114 or 80 mg/kg 4-PBA were stained with PAS (upper) and anti-mucin5AC antibody (lower) showing the quantitation of positive cells. Data are expressed as the mean ± SD and were analysed by ANOVA (n=10). ($^{\#}p < 0.05$ versus saline; $^{*}p < 0.05$ versus *Af*). *Af*, *Aspergillus fumigatus*; IC, IC87114; 4-PBA, 4-phenylbutyric acid; PAS, periodic acid-Schiff.

Supplementary Figure 4

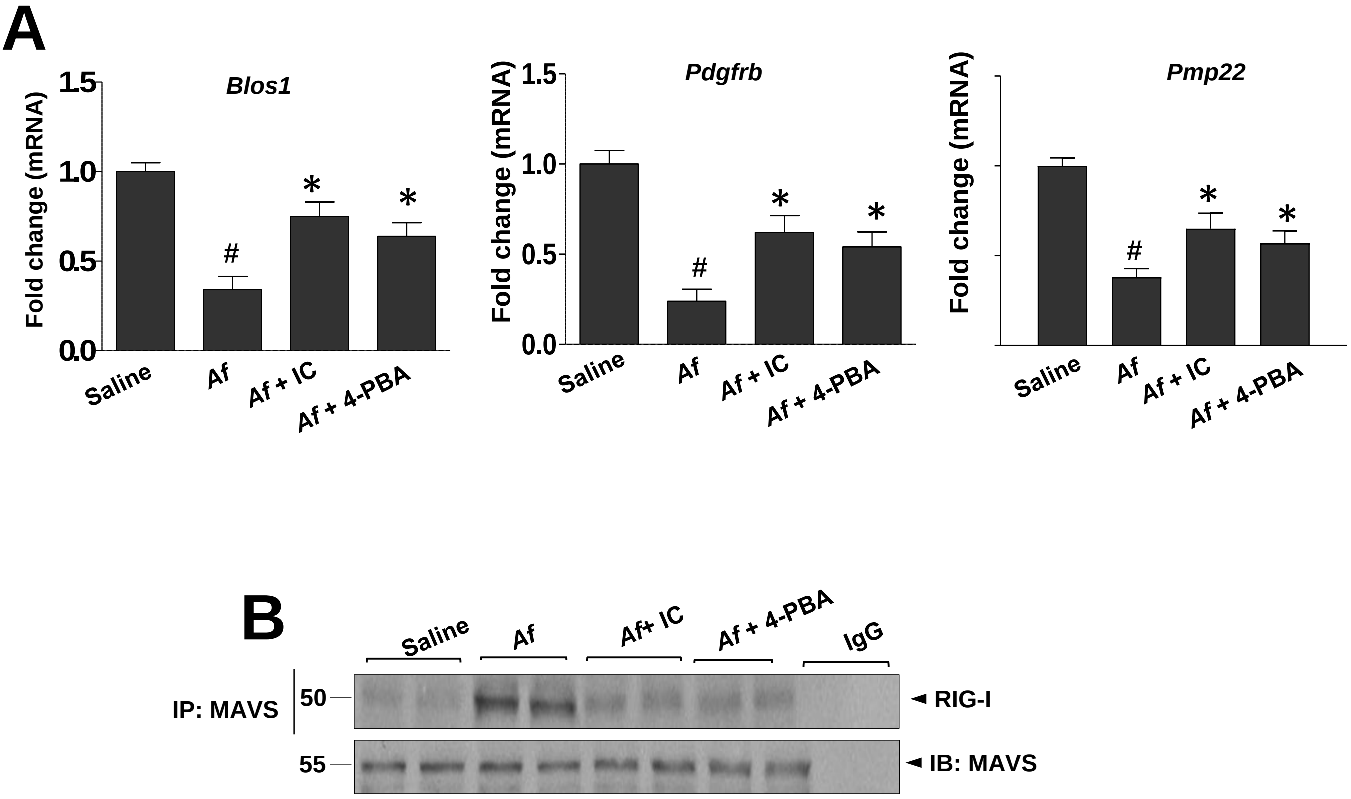

Supplementary Figure 4. IC87114 and 4-PBA alleviates *Aspergillus fumigatus*-induced RIG-1 signaling by controlling RIDD activity. (A) mRNA levels of known IRE1-RIDD target genes *Blos1*, *Pdqfrb*, and *Pmp2*. (B) Lung lysates were subjected to immunoblotting and immunoprecipitation with antibodies against MAVS or RIG-I. Data are expressed as the mean ± SD and were analysed by ANOVA (n=10). ($^{\#}p < 0.05$ vs. saline; $^{*}p < 0.05$ vs. *Af*). *Af*, *Aspergillus fumigatus*; IC, IC87114; 4-PBA, 4-phenylbutyric acid

Supplementary Figure 5

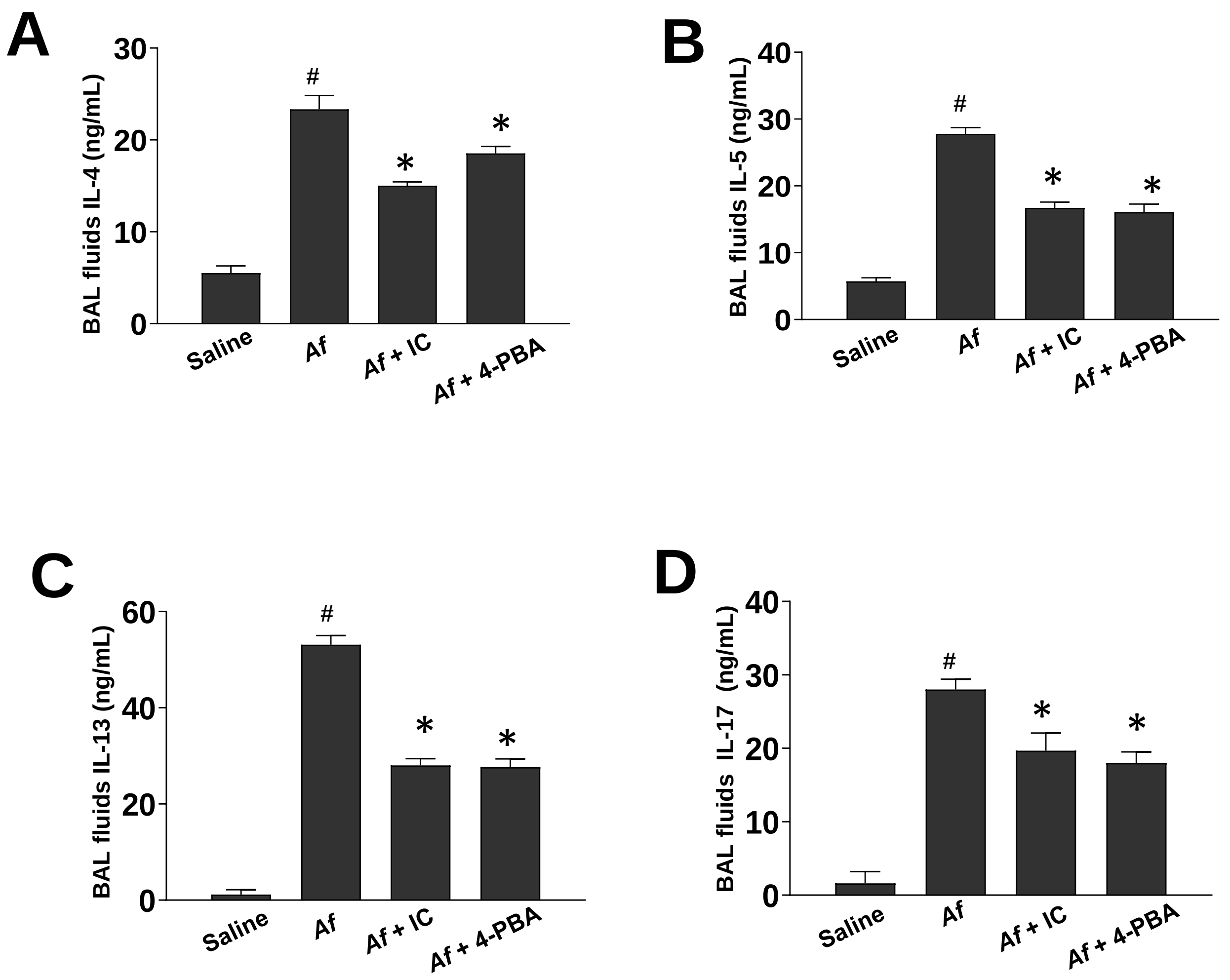

Supplementary Figure 5. IC87114 and 4-PBA reduce inflammatory signaling in lungs and BAL fluids of *Aspergillus fumigatus (Af)*-induced allergic lung inflammation. BAL fluids were collected and analyzed by ELISA for the cytokines IL-4 (A), IL-5 (B), IL-13 (C), and IL-17 (D). Data are expressed as the mean ± SD and were analysed by ANOVA (n=10). ($^{\#}p < 0.05$ vs. saline; $^{*}p < 0.05$ vs. *Af*). *Af*, *Aspergillus fumigatus*; IC, IC87114; 4-PBA, 4-phenylbutyric acid

Supplementary Figure 6

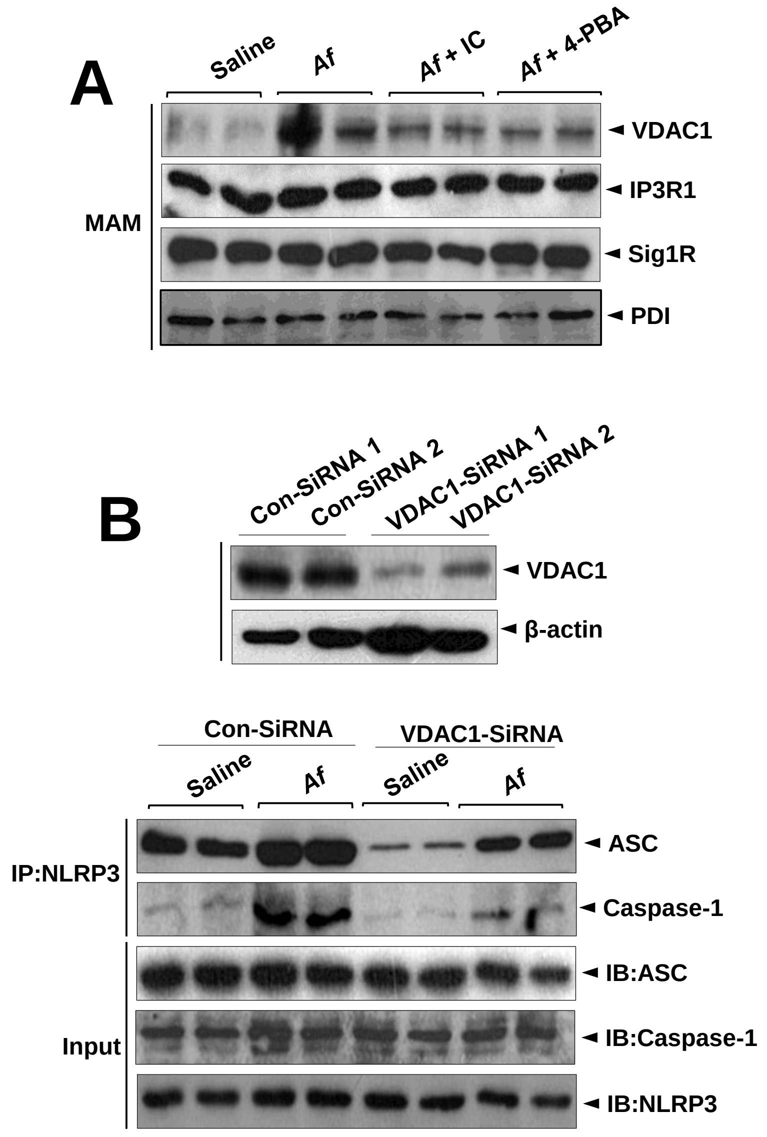

Supplementary Figure 6. VDAC is essential for NLRP3 inflammasome activation. Lung tissues were obtained from *Af*-challenged mice, saline-treated mice, and *Af*-challenged mice treated with 1 mg/kg IC87114 or 80 mg/kg 4-PBA. (A) Immunoblot analysis of VDAC1 (outer mitochondrial membrane marker), IP3R1 (ER marker), Sig1R (mitochondria-associated endoplasmic reticulum membranes marker), and PDI (*ER marker*) in MAM fractions of lung tissue. (B) BEAS-2B cells transiently transfected with non-specific siRNA or stably expressing VDAC1-specific siRNA were subjected to immunoblotting with anti-VDAC1 or β-actin antibody. BEAS-2B cells were treated with 0.1 mg/ml or 5 mM 4-PBA with or without 100 μg/mL *Af* for 24 h. BEAS-2B cell expressing siRNA against VDAC1 were immunoprecipitated with anti-NLRP3 antibody and immunoblotted with antibody against NLRP3, ASC, and caspase-1. MAM, mitochondria associated ER membranes; *Af*, *Aspergillus fumigatus*; IC, IC87114; 4-PBA, 4-phenylbutyric acid

Supplementary Figure 7

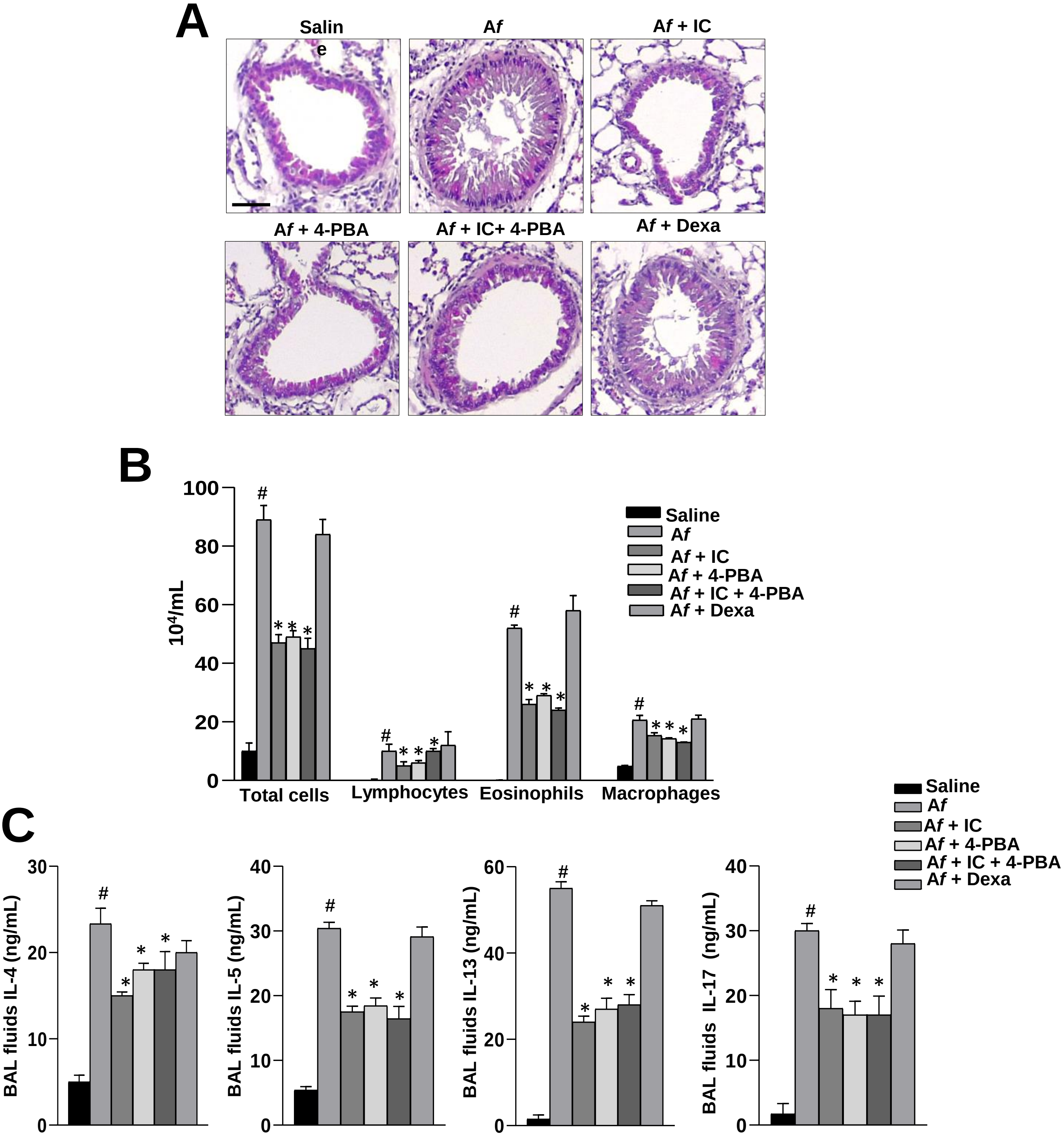

Supplementary Figure 7. Dexamethasone does not affect inflammation in *Af*-induced allergic lung inflammation model. (A) The lung tissues and BALF cells obtained from *Af*- and saline-treated mice and *Af*-challenged mice, treated with 1 mg/kg IC87114, 80 mg/kg 4-PBA, 1 mg/kg IC87114 + 80 mg/kg 4-PBA, or 1 mg/kg dexamethasone, were stained with haematoxylin and eosin. (B) The entire cells and the distinct cellular components of BALF. (C) BAL fluids were collected and the levels of the cytokines (IL-4, IL-5, IL-13, and IL-17) were estimated by ELISA. Data are expressed as the mean ± SD and were analysed by ANOVA (n=10).. $^{\#}p < 0.05$ vs. saline; $^{*}p < 0.05$ vs. *Af*. *Af*, *Aspergillus fumigatus*; IC, IC87114; 4-PBA, 4-phenylbutyric acid
